# Clinical heterogeneity among a three-generation Japanese family with D18N *TREX1* mutation for Aicardi-Goutières syndrome / familial chilblain lupus

**DOI:** 10.1186/1546-0096-9-S1-P276

**Published:** 2011-09-14

**Authors:** J Abe, R Nishikomori, K Izawa, T Awaya, T Kawai, T Yasumi, T Heike, N Hiragi, T Hiragi

**Affiliations:** 1 Department of Pediatrics, Kyoto University Graduate School of Medicine, Kyoto, Japan; 2 Department of Pediatrics, Municipal Tsuruga Hospital, Tsuruga, Japan

## Background

Aicardi-Goutières syndrome (AGS) is a genetic disease, characterized by encephalopathy with cerebral calcification, white matter abnormalities, cerebral atrophy, elevated interferon-alpha in the cerebrospinal fluid and chilblain. Most of AGS patients have severe neurological findings including developmental delay. Five genes, namely *TREX1*, *RNASEH2B*, *RNASEH2C*, *RNASEH2A*, *SAMHD1* have been reported to be responsible for AGS. Most cases of AGS are inherited as autosomal recessive manner, although autosomal dominant AGS is rarely reported and its clinical manifestations are largely unknown.

## Aim

We found a three-generation Japanese family whose members shared severe chilblain in an autosomal dominant manner. We tried to identify the responsible gene and investigated genotype-phenotype correlation within the family.

## Methods

We performed sequencing of the genes responsible for AGS and obtained clinical information from the family.

## Results

The proband had severe chilblain, mental retardation, cerebral calcification, and elevated serum interferon-alpha. We identified a heterozygous *TREX1* mutation, D18N (52G>A) and diagnosed her as AGS. Her mother had the same D18N mutation, although she lacked neurological impairments and cerebral calcification was comparable to the age-matched controls. We diagnosed her mother as familial chilblain lupus (FCL). The proband’s nephew also shared the same D18N mutation with neurological abnormalities including mental retardation and epilepsy. However, he lacked cerebral calcification and chilblain. These data indicated that the same *TREX1* mutation D18N caused AGS, FCL, and the neurological disorder without brain calcification in the same family.

## Conclusion

It should be alerted that autosomal dominant *TREX1* mutation D18N has clinical variation when performing genetic test for AGS/FCL.

